# CASTELO: clustered atom subtypes aided lead optimization—a combined machine learning and molecular modeling method

**DOI:** 10.1186/s12859-021-04214-4

**Published:** 2021-06-22

**Authors:** Leili Zhang, Giacomo Domeniconi, Chih-Chieh Yang, Seung-gu Kang, Ruhong Zhou, Guojing Cong

**Affiliations:** 1 IBM Thomas J. Watson Research Center, 1101 Kitchawan Rd, 10598 Yorktown Heights, NY USA; 2 ZheJiang University, 688 Yuhangtang Road, Hangzhou, 310027 China; 3 Oak Ridge national laboratory, 1 Bethel Valley Rd, 37830 Oak Ridge, TN USA

**Keywords:** Lead optimization, Drug discovery, Molecular dynamics simulation, Machine learning, Variational autoencoder, Clustering

## Abstract

**Background:**

Drug discovery is a multi-stage process that comprises two costly major steps: pre-clinical research and clinical trials. Among its stages, lead optimization easily consumes more than half of the pre-clinical budget. We propose a combined machine learning and molecular modeling approach that partially automates lead optimization workflow in silico, providing suggestions for modification hot spots.

**Results:**

The initial data collection is achieved with physics-based molecular dynamics simulation. Contact matrices are calculated as the preliminary features extracted from the simulations. To take advantage of the temporal information from the simulations, we enhanced contact matrices data with temporal dynamism representation, which are then modeled with unsupervised convolutional variational autoencoder (CVAE). Finally, conventional and CVAE-based clustering methods are compared with metrics to rank the submolecular structures and propose potential candidates for lead optimization.

**Conclusion:**

With no need for extensive structure-activity data, our method provides new hints for drug modification hotspots which can be used to improve drug potency and reduce the lead optimization time. It can potentially become a valuable tool for medicinal chemists.

**Supplementary Information:**

The online version contains supplementary material available at 10.1186/s12859-021-04214-4.

## Introduction

At a time of global health crisis, drug discovery is of utter importance to bring the society back to its order. Beyond the crisis, drugs have helped to improve life quality and increase the life expectancy. However, despite the growing research and development expenditure every year [1, 2], the yearly FDA-approval of drugs has mostly stalled since 1993 [3]. In fact, there were a total of 3437 FDA approved small-molecule and large-molecule drugs or therapeutics in 2018 [4], with a yearly addition of only $\sim$ 1.2% (2014–2018 average). The so-called “Eroom’s law” overshadows the pharmaceutical industry, with new molecular entity (NME) drugs per billion dollars spent decreasing since 1950 [5]. While millions of people globally are suffering from incurable diseases such as Parkinson’s disease [6], Huntington’s disease [7], HIV/AIDS [8] and hepatitis B [9], technological revolutions are required to find the cures and possibly avert Eroom’s law.

Computer-aided drug discovery (CADD) methods have been widely used in the pharmaceutical industry, from quantitative structure-activity relationships (QSAR, [10]), pharmacophore modeling [11] to drug-target docking algorithms [12]. Along with the conventional high-throughput screening (HTS [13]) method and rising fragment-based drug discovery (FBDD [14]) method, computational methods have helped to lower the initial cost of drug discovery (target-to-hit and hit-to-lead, specifically). The resulting lead products usually have a dissociation constant ($K_\text {d}$) in the micromolar range. The subsequent process would aim to optimize the drug lead by lowering the $K_\text {d}$ value to the nanomolar range while ensuring the safety of the drug (and therefore termed “lead optimization”). However, the optimization processes highly rely on domain knowledge and luck because of the astronomical possibilities in the chemical space. Several computational methods have been developed to assist lead optimization. For example, a semi-automated optimization method was suggested by Lewis, combining 3D-QSAR model, random structure permutation methods and human decisions [15]. Jain applied a combined QSAR-docking method to an existing library of CDK2 inhibitors and showed encouraging agreement with the experiments [16]. Tang et al. improved zinc endopeptidase inhibitor by intuitively suggesting drug modifications from a binding mode suggested by the molecular dynamics (MD) simulation [17]. Jorgensen et al. adopted Monte Carlo (MC) simulations and free energy perturbation (FEP) simulations to virtually screen chemical groups on a drug lead [18]. However, despite these developments, lead optimization remains the most costly step before the clinical trials, constituting $\sim$ 74% of the pre-clinical costs [19]. A common issue of the previous computational methods lies in the generation of new structures, which mostly relies on time-consuming systematic exploration of substitutions at each location in the molecule. Therefore, an efficient way of identifying improvable molecular fragments is needed to assist lead optimization decisions. Notably, computer-aided FBDD algorithms [20] can suggest leads by combining the fragment hits (fragments with relatively strong affinity, typically with $K_\text {d}$ < 10 mM [21]). But the use is limited once the lead has been identified.

**Fig. 1:**
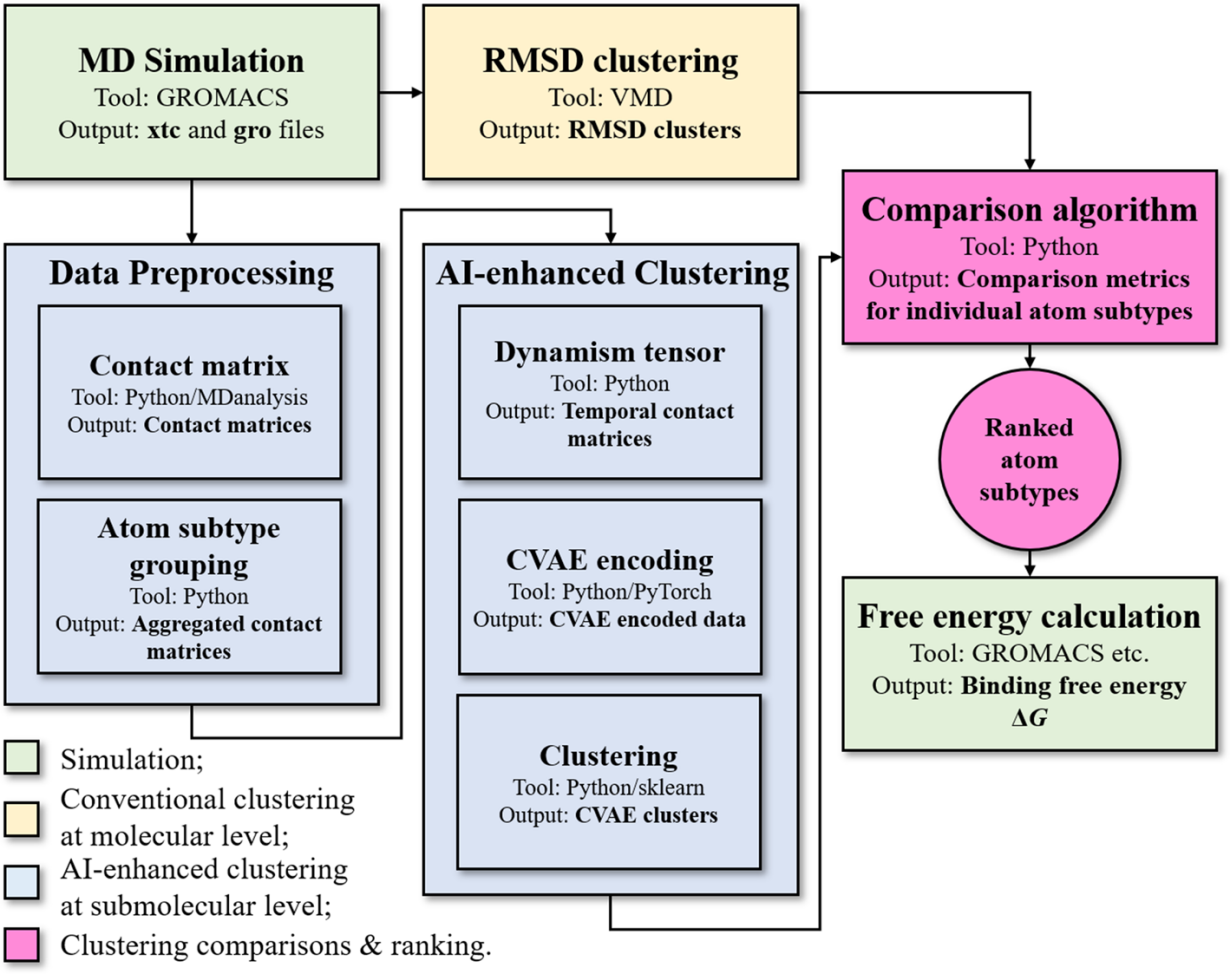
The general pipeline for CASTELO. The starting point is the generation of MD trajectories, with tools such as GROMACS. RMSD clustering can be done with VMD software. In another route, we process MD trajectories with python scripts to obtain contact matrices. Atom subtype information is used to aggregate the calculated contact matrices. Following that, dynamism tensors with temporal information is generated on top of the contact matrices using python scripts. CVAE model is used to encode the dynamism data, before clusters are calculated with tools such as HDBSCAN. Finally, we converge the two routes by comparing clusters from conventional RMSD clustering and CVAE clustering with proposed comparison metrics. The atom subtypes are ranked, as the final output of CASTELO. With domain knowledge, we suggest modifications for the lowest ranked atoms. Methods such as free energy perturbation calculations can be used to verify CASTELO’s suggestions

In this study, we propose a computational method, coined Clustered Atom Subtypes aidEd Lead Optimization (CASTELO), that identifies modifiable submolecular moieties in a lead molecule to narrow down the substitution sites to a few possibilities. The process is depicted in Fig. 1. Briefly, we first obtain the target-lead binding complexes from either crystal structures or reasonable computational methods (such as homology modeling, docking and MD simulations). Using this structure, $\sim$ 100 ns of MD simulations are then conducted. Subsequently, contact matrices, containing the relative distances between atoms in the lead and atoms in the target protein, are extracted from the simulations. The contact matrices are processed to contain temporal information (coined “dynamism tensor”) and atom subtype information (see details below). Compressed vectors are then created with convolutional variational autoencoder (CVAE) using the processed contact matrices. CVAE automatically reduces the high dimensionality of the contact matrix into a latent space where states that share similar structural and energetic characteristics [22] to each other. In order to consider the dynamic behavior of the target and the lead, each time step is modeled with a dynamism tensor (a 2-dimensional contact matrix) that contains target-lead interaction information from the MD simulation. This tensor, closely resembling as a 2-channel image, is then fed to the CVAE. The latent space representation generated by the CVAE allows the clustering of the time steps of the MD simulation, grouping time steps with similar behavior. Stable snapshots will be clustered in big clusters, while unstable ones will generate a number of small clusters [23–25].

The goal of our method is to discover specific submolecular moieties of the lead molecule that harm the target-lead interactions (thus termed as “malicious atoms”). One way to group the atoms in a molecule to “submolecular moieties” is to categorize them in subtypes based on their physical properties (see Method for details). To differentiate the contributions of the subtypes, CVAE and clustering are not only applied on the whole dynamism tensors, but also to the tensors of each subtype. Clustering information is thus generated for each subtype and compared with conventional clustering information (such as from root mean sqaure displacement (RMSD) clustering) of the whole molecule. Finally, using comparison metrics such as *cosine similarity (CosSim)* or average difference (see details below), we rank the subtypes from malicious atoms to beneficial atoms. If the overall simulation renders a stable binding structure for the lead (which is often the case), the atom subtypes with lower values of comparison metrics are labeled as malicious atoms, i.e. modifiable atoms for lead optimization, because of their “deviation” from the stable binding state of the rest of the molecule. To verify if these atoms are indeed improvable, we modify the suggested atoms and calculate binding free energy change using FEP calculations.

**Fig. 2:**
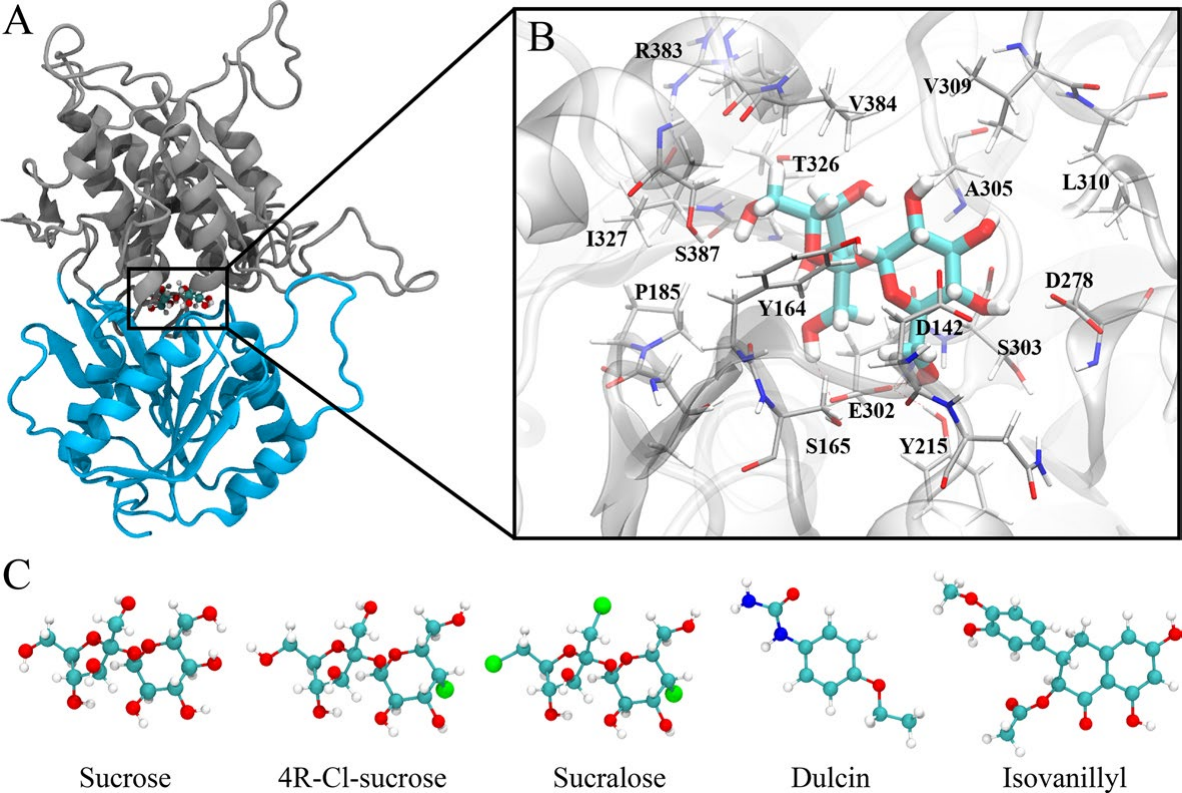
The structure of sucrose bound T1R2 is shown in subplot** a** and** b**. The T1R2 structure is drawn in two colors (gray and blue) just to highlight its venus-flytrap-like structure. The detailed interactions between sucrose and its surrounding residues are depicted in **b**. The structures of the five sweeteners tested in this study are illustrated in **c**

As an example, we applied this process to the sweetness taste receptor T1R2 with five well-known sweeteners: sucrose (the reference for relative sweetness, RS = 1 [26]), (1R,2R,3R,4R,5R)-4-Chloro-1-[(2R,3S,4S,5R)-3,4-dihydroxy-2,5-bis(hydroxymethyl)oxolan-2-yl]oxy-6-(hydroxymethyl)oxane-3,4-diol (4R-Cl-sucrose thereafter, RS = 5 [27]), sucralose (RS = 600 [26]), dulcin (RS = 250 [26]), and an isovanillyl sweetener (isovanillyl thereafter, RS = 400 [28]). Note that because of the conceptual similarity between drug potency and sweetener potency, drug discovery methods such as systematic substitutions have been often seen in improving sweeteners [27, 28]. To model the sweeteners with MD simulations, the structure of sweetness taste receptor T1R2 is taken from Perez-Aguilar et al. [29], shown in Fig. 2a. The sweetener molecules are shown in Fig. 2c. The search of the binding modes for all five sweeteners follows the the combined docking-MD method reported previously [30]. With CASTELO, we are able to suggest modifications that improve molecular sweetness of the simulated molecules by identifying malicious atoms. Importantly, no structure-activity data are needed for this process. The minimal requirement is a lead molecule and a target protein structure.

## Methods

### Molecular docking

The protein structure of T1R2 was taken from Perez-Aguilar et al. [29]. The structures of sucrose, 4R-Cl-sucrose, sucralose, dulcin and isovanillyl were constructed with Jmol [31]. We referred to previous studies [32] and assumed that the “flytrap” domain (referring to the boxed region in Fig. 2a due to the resemblance between T1R2 and the venus flytrap) of T1R2 should be the binding domain for sweeteners. Initial binding structures were then constructed by selecting the “flytrap” domain in T1R2 using Autodock Vina [33].

**Fig. 3:**
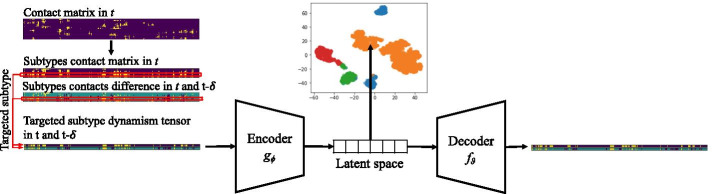
CVAE pipeline. Left part of the Fig. shows the input preparation: the contact matrix of a time step *t* is grouped over the *y*-axis by drug atom subtypes (yellow cells are contacts). Each contact matrix of a time step *t* is paired to its temporal difference with a previous one $t-\delta$ to form a dynamism tensor (violet/yellow cells are new/disappearing contacts, green cells are stable). Each atom subtype is selected in turn and fed to the CVAE. The representation in the latent space is used to cluster the time steps by similar behaviors

### Molecular dynamics simulation (dataset)

The initial binding structures constructed from Autodock Vina were solvated with water in a simulation box of 10 $\times 10 \times$10 nm$^3$. 0.1 M of NaCl was then used to ionize and neutralize the simulation box. The resulting simulation systems contained roughly 105,000 atoms. CHARMM36 force field [34] was used for the T1R2 protein, water and ions. CGenFF force field was generated with the online server [35] for the five sweeteners. NAMD2.9 [36] was used to run all simulations on Blue Gene supercomputer. Particle mesh Ewald (PME) was used for electrostatic interaction calculations with a grid size of 1 Å. A switching function was used for van der Waals (VDW) interactions calculations, where switching distance was 10 Å, cutoff distance was 12 Å and VDW pairlist distance was 13.5 Å. Before production runs, 20,000 steps of energy minimization and 250 ps of equilibration using 0.5 fs time step were performed. For the production runs we used 2 fs as the time step. Depending on the stability of the structures, 50 to 100 ns of simulations were performed for each of the docked structures. Snapshots of the trajectories were saved every 20 ps for the data collection. Overall, 11 simulations were conducted (2 for sucrose, 1 for 4R-Cl-sucrose, 1 for sucralose, 2 for dulcin and 5 for isovanillyl). RMSD clusering method [37] was used to determine the stability of the binding modes in these simulations. 5 simulations (1 simulation per each sweetener) were chosen as “stable simulations” (more details below). All simulations were used to train machine learning models.

### Free energy perturbation (FEP) calculations

Following previous studies [25, 30], we used Zwanzig equation [38] and thermodynamic cycle shown in Additional file 1: Fig. S1 to calculate the relative binding free energy of the five sweeteners with “muted ethane” (refered to as ethane thereafter for simplicity) as the reference point. This ethane construction had the structure of ethane but was chargeless. We performed two sets of simulations with NAMD2.9 for each sweeteners (the thermodynamic cycle is shown in Additional file 1: Figure S1): the “bind” simulations where T1R2 was present and sweeteners were mutated to ethane; and “free” simulations where T1R2 was absent and sweeteners were mutated to ethane. The resulting free energy change was denoted as ${\Delta }F_{bind}$ and ${\Delta }F_{free}$. The relative binding free energy of the sweeteners can then be calculated as: ${\Delta \Delta }F_{SWT} = {\Delta }F_{SWT} - {\Delta }F_{ethane} = {\Delta }F_{free} - {\Delta }F_{bind}$. Assuming that the relative sweetness of the sweeteners was a direct measurement of the dissociation constant $K_d$, we could compute the relative sweetness as follows:

1 $$\begin{aligned} CRS(SWT) = e ^ {- ({\Delta \Delta }F_{SWT} - {\Delta \Delta }F_{sucrose})/RT } \end{aligned}$$

**Fig. 4:**
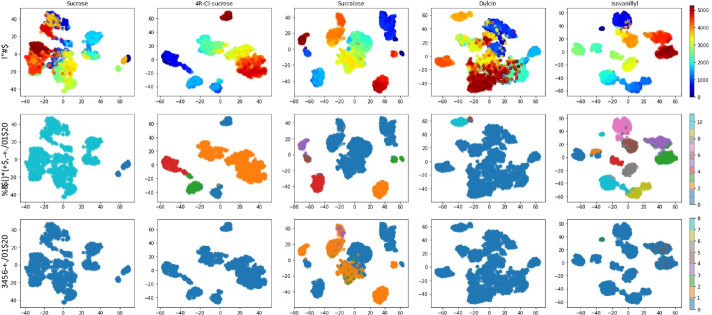
T-SNE projection of the CVAE latent representations. The top row uses a color map based on the simulation time. The middle and bottom rows use colors to distinguish clustering results respectively of our AI-enhanced model and of the RMSD. Here we depict the encoded representations generated by the CVAE model with $f=32$ filters and $d=5$ latent dimensions. The calculated cosine similarity values are 0.975, 0.871, 0.785, 0.960 and 0.882 between AI-enhanced cluster sizes and RMSD cluster sizes for five sweeteners from left to right, respectively

### Grouping submolecular moieties using atoms subtypes

We categorized atoms using the VDW parameters of the atom types (such as CG331) from the CHARMM36 force field. Atom types that fell in the range of 10% of $\sigma$ (particle size) and $\epsilon$ (dispersion energy) with each other were classified into the same “atom subtype”. Such classification resulted in 46 atom subtypes from the approximate 300 atom types from the CHARMM36 protein, carbohydrate and cgenff force field. The 46 atom subtypes were used to differentiate “beneficial atoms” (atom subtypes that likely strengthened sweetener-T1R2 interactions) from “malicious atoms” (atom subtypes that likely weakened sweetener-T1R2 interactions). Note that we chose these the atom subtypes based on our experience with the current dataset. Other subtyping methods are also possible and will be explored in future studies.

### AI-enhanced clustering

Autoencoders are unsupervised neural networks widely used for dimensionality reduction and pattern recognition. Their architecture is composed of an encoder part $g_\phi$ that compresses an input into a latent space and a decoder part $f_\theta$ that reconstructs the original input using the low-dimensional features. Variational autoencoders (VAEs) introduce the optimization constraint to the latent space to be normally distributed, this coerces the network to distribute the information more evenly into the latent space [39]. In MD simulation analysis, a common input is a contact matrix from a single MD time step; hence, convolutional layers (CVAE) rather than regular feedforward are typically used. This leads to filter maps that can better recognize local patterns independently of the position.

We used CVAE to model each MD trajectory in a low-dimensional space. Similar to previous works [22, 40], we modelled the input as a contact matrix between atoms of the drug molecule and atoms of the protein in each structure of the simulation. Specifically, the contact matrix has size of $N \times M$, where N is the number of drug atoms and M is the number of protein atoms. Our goal was to find binding patterns during the simulation, hence the temporal information over time was important to locate stable and unstable states. We enriched each time step *t* input with a contacts’ dynamism representation: the difference of contacts between the current time *t* and a previous one $t-\delta$, where $\delta$ is a parameter of our framework. To maintain the dynamism matrix binary AND indipendent to the direction of the movement, we used the absolute value of the difference. In this matrix, 0 cells represented a stable situation, while 1 cells showed a dynamic behavior (either a new contact or the disappearance of a previous contact). Each time step of the simulation was then modeled as a tensor of $2 \times N \times M$ (termed as the dynamism tensor), with the two elements of the first dimension representing respectively the contact matrix in *t* and the temporal difference of the contacts in *t* and $t-\delta$. We used $\delta =500$ (i.e. 10 ns) in our experimental setting.

CVAE represents each contact tensor in a *d*-dimensional latent space, i.e. a vector of *d* elements. This intrinsically brings time steps with similar contact matrices to closer positions in the latent space, allowing clustering techniques to group sets of time steps with similar behavioral patterns. In other words, applying clustering over the latent space vectors allows the discrimination of big clusters with several time steps of similar behavior, i.e. stable situation, or small clusters, i.e. unstable. In CASTELO, we used HDBSCAN [41] as our clustering method. This algorithm overcame the limitation of knowing in advance the number of clusters, that was the typical drawback of partitioning techniques such as K-means, and density threshold, a typically required by the standard DBSCAN.

The procedure described above would provide *general* binding information of a MD simulation, i.e. states of the whole drug molecule. CASTELO could provide a fine-grained perspective of the drug molecule behavior: a specific binding view of each atom, or group of atoms. For this purpose, we grouped the drug atoms according to their physical properties (as described in previous Section) and we applied the CVAE and clustering pipeline described above by only focusing on the contacts of each individual atom subtype. In this case, each time step was sampled by a tensor of size $2 \times 1 \times M$ (*N* became 1 for each individual atom subtype), which becomes a 2-channel image of size $2 \times M$ by squeezing the *N*-dimension. Figure 3 shows the pipeline with the input preparation of one targeted atom subtype. We repeated the CVAE and clustering procedure for each atom subtype of the drug. By differentiating the individual atom subtypes, this approach allows the selection of atoms that behave differently from the overall binding behavior (detailed in next section).

**Fig. 5:**
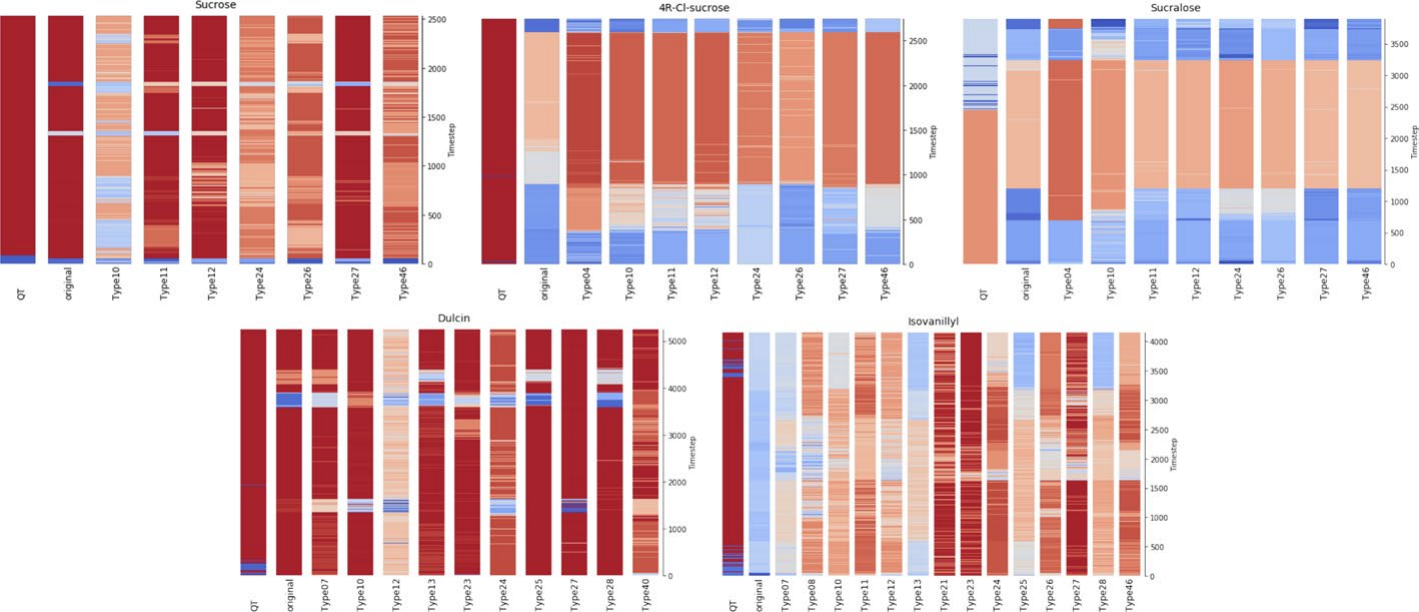
Clustering results for all atom subtypes in the five sweeteners. The first bar shows the RMSD clustering with VMD1.9.3. The second bar is calculated with CVAE and HDBSCAN when all atoms are considered. The following bars represent the clustering results for each atom subtype of the corresponding sweetener. Each plot depicts the cluster size at time *t* against simulation time *t*. For CVAE and HDSCAN results, each cluster size *t* is the average of the sizes given by the 6 trained models. Bigger clusters are colored with dark red while small clusters are colored with dark blue

CASTELO pipeline was completely formed by unsupervised techniques, that were hard to tune and evaluate. In order to provide reliable suggestions, we trained slightly different CVAE architectures varying the hyper-parameters. In our experiments, we varied the latent dimensions *d* and the number of convolutional filters *f*. This choice was made because we noticed few cases of all non-clustered samples when latent space dimension *d* is too big. We also tested the results by varying the time $\delta$ of the contacts’ dynamism, but the contribution was not pronounced.

### Experimental settings

As described above, we trained a number of CVAE models for each atom subtype. Each input sample, i.e. a time step *t*, is represented by a $2 \times M$ tensor selecting the contact and dynamism vectors related to the targeted subtype. In our experimental settings the CVAE encoder part was formed by four convolutional layers with *f* filters of size 1x7, we decided to keep the convolutions between contacts and dynamism separated, thus the 1-sized filter in the first dimension. We trained models varying the filter numbers $f \in \{32,64\}$. Considering the highly narrowed shape of the input, we used a stride of 2 in the second dimension, i.e. on the protein atoms. We did not use pooling or padding. The decoder part was specular to the encoder. We used three different values for the latent space size: $d \in \{3,5,10\}$. For each atom subtype we thus trained 6 different CVAE architectures, varying *f* and *d*. We combined BCE and KLE as loss function [42], using RMSProp as optimizer and a learning rate of 0.005. We trained each CVAE model for a maximum of 600 epochs with an early stopping mechanism after 10 epochs without improvement in the loss. We set HDBSCAN with a minimum cluster size of 50, all the other parameters were set to the default values.

### Submolecular moiety suggestion for lead optimization

The suggestion of malicious atoms to increase the binding affinity of the molecule was made by a comparison of the clustering result of each atom subtype with respect to the clusters obtained while considering the whole molecule. In this comparison, a clustering that considered the whole molecule was needed as reference. A CVAE model that had the contact matrices with all the drug atoms as input might be used as reference. Otherwise, in order to avoid possible similarity biases due to the same architecture used by the compared clusters, any other traditional clustering methodology could be used as reference. In our experiments, we decided to use the default RMSD clustering provided by VMD1.9.3. [37]

As described in the previous section, each atom subtype had a number of clustering results, one for each trained CVAE architecture. The final comparison was made with the averaged values of the comparison of each architecture with the whole molecule. This comparison also provided a standard deviation value that might be used as agreement score of the different CVAE models of the same atom subtype. I.e. averaged comparison values with small standard deviation indicated a well grounded suggestion.

More specifically, two types of comparison metrics were explored in this study, with the first one being the cosine similarity (*CosSim*):

2 $$\begin{aligned} CosSim = \frac{ \sum \limits _{t \in T} C_{t,A} \cdot C_{t,S} }{ \sqrt{ \sum \limits _{t \in T} C_{t,A}^2 } \cdot \sqrt{ \sum \limits _{t \in T} C_{t,S}^2 } } \end{aligned}$$

where *t* is a time frame that belongs to the trajectory (*T*), $C_{t,A}$ is the cluster size of the cluster at time *t* for atom subtype A, calculated with the AI-enhanced clusering, $C_{t,S}$ is the cluster size of the cluster at time *t* for the whole molecule (S), calculated with conventional methods such as RMSD clustering. Note that *CosSim* has a range of [0, 1], with lower values indicating deviation between the two arrays and higher values indicating similarity.

The second comparison metric examined was average difference (AvgDiff):

3 $$\begin{aligned} Avg\,Diff = \frac{ \sum \limits _{t \in T} ( C_{t,A} - C_{t,S} ) }{ \text {count}(T) } \end{aligned}$$

where $\text {count}(T)$ is the total number of time frames for the trajectory. The values of *AvgDiff* have a wider range than *CosSim*, depending on the cluster sizes. Generally speaking, a negative *AvgDiff* indicates that subtype A is less stable than the whole molecule (S), while a positive *AvgDiff* indicates the opposite.

The two comparison metrics were then normalized by the average values over all subtypes: $nCosSim = CosSim - Avg(CosSim)_{subtypes}$; $nAvgDiff = AvgDiff - Avg(AvgDiff)_{subtypes}$. After assigning a normalized comparison metric value, each of the atom subtypes was given a final ranking, with the lowest ranked subtype as the most “malicious”, and therefore the top suggestion for lead optimization. As a follow-up in this study, we used domain knowledge to suggest some modifications and tested the modifications with FEP calculations (see results for more details).

## Results and discussion

### RMSD clustering identified stable binding mode and binding motifs

MD simulations were used to identify stable binding modes of the sweeteners in the binding pocket of T1R2 flytrap domain (shortened for T1R2 thereafter) of human sweetness taste receptor [32]. We prepared the initial guesses of the binding structures using molecular docking software Autodock Vina [33]. One to five highest scored docking result(s) were taken as the starting structures in molecular dynamics simulations. Upon the completion of 50–100 ns of simulations, binding mode clusters were classified using RMSD clustering method provided in VMD [37] using 2.0 Å as the cutoff. RMSD values were calculated on the sweetener molecules only, while T1R2 structures were aligned. The identified clusters (binding modes) were considered as “stable” if the cluster size persisted longer than 50 ns, following the practices in previous studies [25, 30]. The persistent time of the clusters for all five sweeteners were plotted in Additional file 1: Figure S2 (only the trajectories with the largest clusters were shown), indicating that stable binding modes were found for each of the sweeteners.

**Fig. 6:**
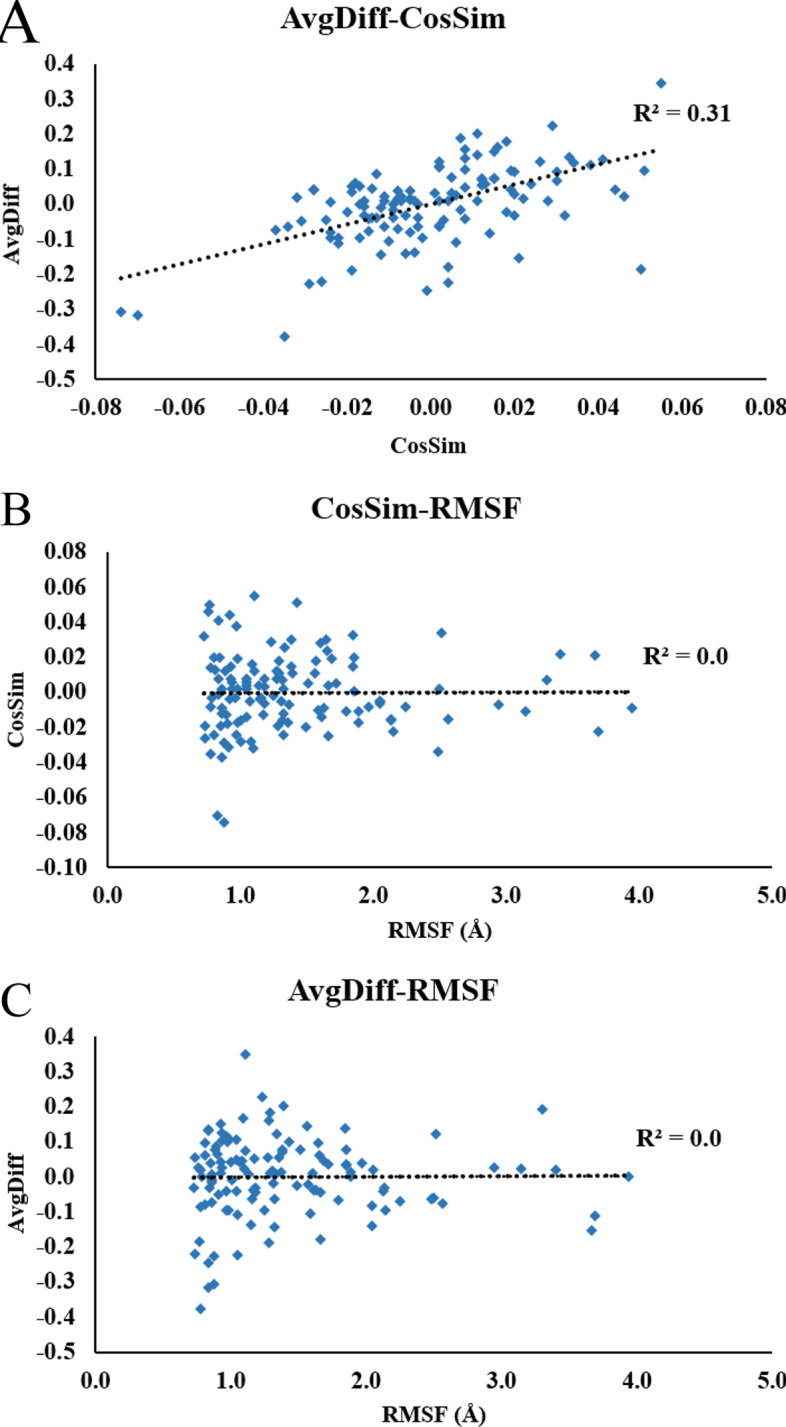
Comparison metrics are calculated by comparing the clusters with conventional RMSD clustering and AI-enhanced clusering with Eqs. 2 and 3. In **a** we show that the two comparison metrics *nCosSim* and *nAvgDiff* (normalized metric *nmetric* is calculated as $metric -Avg(metric)_{subtypes}$) are positively correlated. In **b**, **c** we calculated the RMSF of the atom subtypes for all T1R2-sweetener trajectories. Clearly, *nCosSim* and *nAvgDiff* are not correlated with RMSF

In Fig. 2a, we illustrated the stable binding mode extracted from the T1R2-sucrose simulation. The sucrose molecule resided well within the binding pocket (the flytrap domain of T1R2). A closer look (Fig. 2b) revealed that the crucial binding residues are mostly hydrophilic, including the hydrogen bond receptors D142 D278, E302, among other residues such as Y164, S165, P185, Y215, S303, A305, V309, L310, T326, I327, R383, V384 and S387. If the two loop domains on the right of the boxed region in Fig. 2a were considered as the “lips” of the flytrap, we noticed that sucrose bound deeply in the “mouth”. One indication of the sweetener being closer to the “lips” would be lower residue numbers among the interacting residues (see Additional file 1: Figure S3A for a reference of residue numbering). These findings agreed well with the previous binding structure reported by Zhang et al. [32], where the binding pocket facilitated hydrogen bonds between the 8 hydroxyl groups in sucrose and hydrophilic residues in T1R2. Such hydroxyl group-facilitated sweetness was suggested more than half a century ago and has remained a heavily investigated topic [43–45].

**Fig. 7:**
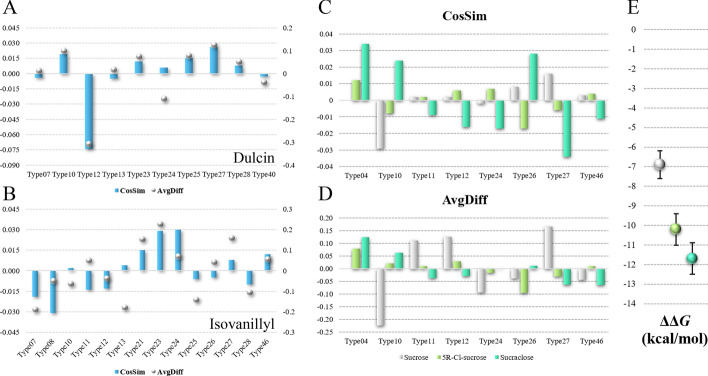
We rank the atom subtypes from the five sweeteners with the calculated comparison metrics: *nCosSim* and *nAvgDiff*. Negative values mean that the corresponding atoms are “malicious”. Positive values mean that the corresponding atoms are “beneficial”. The results for dulcin and isovanillyl are plotted separately in **a**, **b**. We group sucrose, 4R-Cl-sucrose and sucralose due to their structural similarity. The normalized values of comparison metrics of the 8 atom subtypes shared among these three sweeteners are plotted in **c** and **d**, respectively. The relative binding free energy (**e**, with ethane as the reference point) is plotted in juxtaposition to the comparison metrics

The stable binding structures of the rest of the five sweeteners were illustrated in Additional file 1: Figure S3. We found that the crucial binding residues for 4R-Cl-sucrose included D142, E302, Y103, N143, Y164, S165, A166, T184, Y215, S303, W304, I306, V309, L310, I325, T326, I327, R383, and S387, highly similar to those of sucrose. This indicated that the binding mechanism mainly remained the same after changing one hydroxyl group of the sucrose to the chlorine atom in 4R-Cl-sucrose. In contrast, the crucial binding residues for sucralose (D142, E302, L41, I67, L71, S144, Y164, S165, A166, I167, T184, H190, S303, A305, V309, and V384) included more residues on the slightly more hydrophobic “lips” region of the flytrap. This shift was expected because 3 relatively more hydrophilic hydroxyl groups of the sucrose were modified to 3 chlorine atoms in sucralose. Nevertheless, D142 and E302 remained crucial as hydrogen bond receptors for sucralose.

**Fig. 8:**
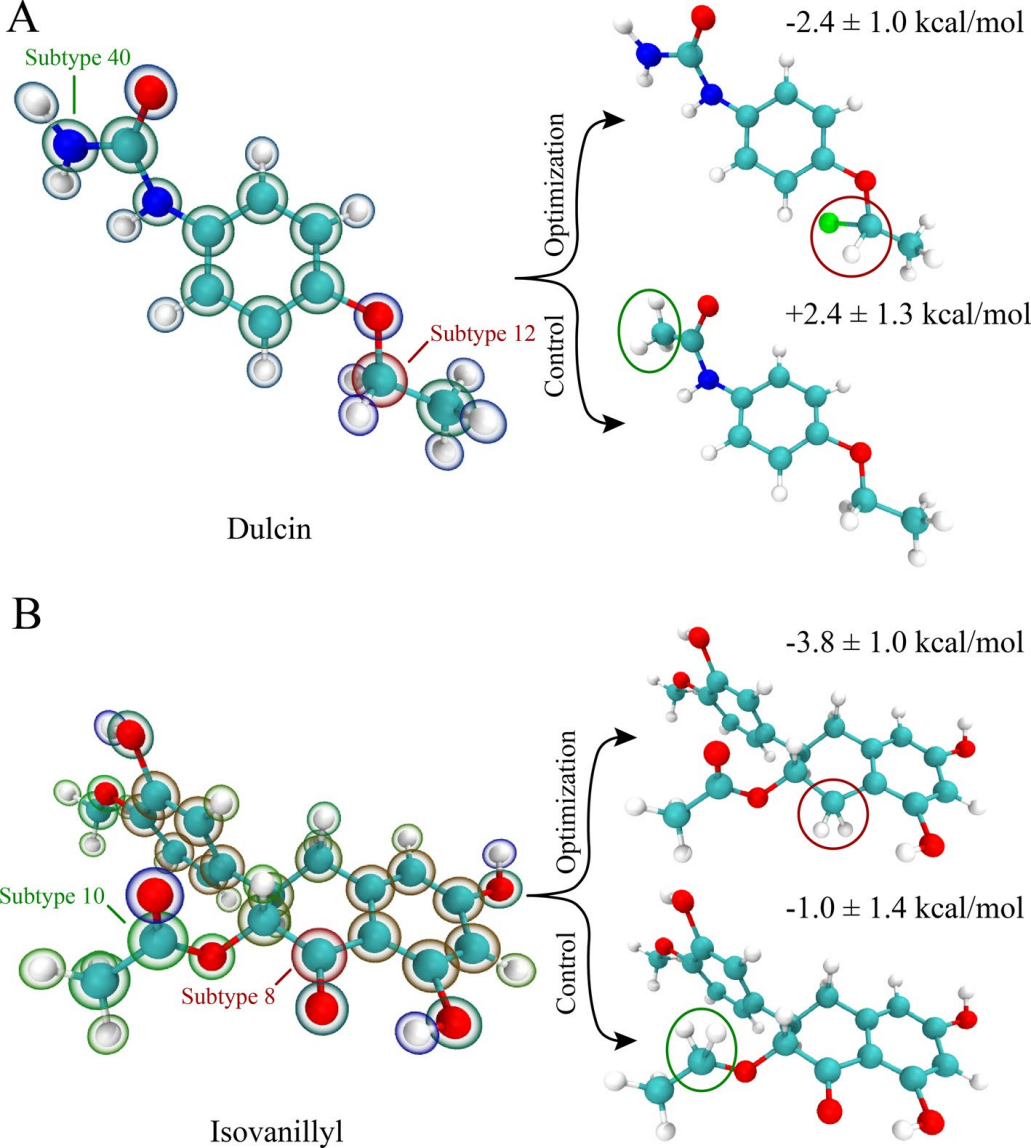
Attempted lead optimization for dulcin (**a**) and isovanillyl (**b**). The molecules are illustrated with two representations: solid stick and ball model for atom identification (cyan for carbon, white for hydrogen, blue for nitrogen, red for oxygen and green for chlorine); bubbles for *nCosSim* values (blue for positive/beneficial, green for neutral and red for negative/malicious). Two examples are illustrated, one for optimization and one as control, for both dulcin and isovanillyl. The resulting binding free energy changes are listed next to the examples, with negative values meaning strengthened binding affinity and positive values meaning weakened binding affinity. A complete list of the lead optimization can be found in Additional file 1: Table S2 and Table S3

The stable binding structure of dulcin interacted with mostly hydrophilic residues L41, Y103, D142, Y164, S165, T184, P185, H190, E302, S303, A305, I306, T326, I327, R383, V384, S387, and L448, similar to those of sucrose and 4R-Cl-sucrose. The main hydrogen bonds appeared to be between the urea group of dulcin and D142. Finally, the stable binding structure of isovanillyl interacted with F39, S40, L41, V64, I67, L71, Y103, D142, Y164, S165, Y215, E302, S303, V309, V384, V385, S387, V388, indicating a binding domain that was more hydrophobic and similar to sucralose. Only occasional (and weak) hydrogen bonds were found between isovanillyl and E302, along with backbone carbonyl groups of several residues.

### Binding free energy of the sweeteners agreed well with experimental relative sweetness

Starting from the stable binding structures of the five sweeteners in this study, we performed FEP calculations for each by constructing “dual topology” between muted ethane and the corresponding sweetener (see Methods for more details). The computed binding free energy ${\Delta \Delta }F$ was therefore the relative binding free energy between the sweetener and muted ethane in T1R2 binding pocket. We assumed that muted ethane had a flat binding energy surface in the T1R2 binding site due to its chargeless nature. Then the calculated binding free energy should be relatively accurate among the five sweeteners. Note that similar approaches have been verified in a number of previous publications [25, 30, 46–48].

We noticed that the general trend of ${\Delta \Delta }F$ values and experimental values of *RS* agreed well with each other (Table 1). The binding mechanisms of the five sweeteners were, however, vastly different as suggested by the interacting residues listed above. To bridge between our speculations on the structural information and quantitation, we reported the electrostatic and VDW contributions in ${\Delta \Delta }F$ by performing energy decomposition for all five FEP calculations (see Additional file 1: Table S1). We found that sucrose, 4R-Cl-sucrose, and dulcin mostly utilized polar interactions. In contrast, isovanillyl mostly utilized non-polar interactions, agreeing well with the structural speculations. Surprisingly, despite being in a similar binding domain to isovanillyl, sucralose still utilized mostly polar interactions. Comparing the binding modes between sucrose, 4R-Cl-sucrose and sucralose, it was likely that the replacement of hydroxyl groups to chlorine lowered the penalty of the remaining hydrophilic hydroxyl groups in hydrophobic environment, rather than directly boosting the hydrophobic interactions.

**Table 1:** Free energy

Sweetener	log(RS)^a^	${\Delta \Delta }F$^b^	log(CRS)^c^
Sucrose	0 [26]	− 6.9 ${\pm }$ 0.7	0 ${\pm }$ 0
4R-Cl-sucrose	0.70 [27]	− 10.2 ${\pm }$ 0.8	2.33 ${\pm }$ 0.76
Sucralose	2.78 [26]	− 11.7 ${\pm }$ 0.8	3.38 ${\pm }$ 0.76
Dulcin	2.40 [26]	− 10.6 ${\pm }$ 0.4	2.61 ${\pm }$ 0.59
Isovanillyl	2.60 [28]	− 11.1 ${\pm }$ 0.8	2.96 ${\pm }$ 0.74

^a^Relative sweetness

^b^Binding free energy relative to ethane (kcal/mol)

^c^Computed relative sweetness is calculated with Eq. 1 in reference to sucrose

We further computed the relative sweetness (*CRS*), listed in Table 1. The computed results agreed generally well with the experimental *RS* values. Such agreement showcased that FEP method could be used to calculate the *RS* of artificial sweeteners. In later sections, we used FEP calculations to validate that “lead optimization” of the sweetener (meaning to increase the *RS* value) was possible using CASTELO.

### AI-enhanced clustering with all atoms agreed well with the RMSD clustering

We firstly tested the reliability of our AI-enhanced clustering with a direct comparison to a reference clustering using RMSD in VMD1.9.3. In order to get a real comparison, we trained the CVAE models using all the sweetener atoms, i.e. with the same contact matrices used by RMSD as input. To have a 2-dimensional tensor as input of the CVAE, we concatenated the contacts and dynamism tensors in the first dimension, forming a $2N \times M$ input. We noticed that the obtained clustering results agreed well with those obtained by RMSD. Figure 4 shows the t-SNE [49] visualization of the latent representations by the CVAE models of each sweetener, each data point is a time step of the simulation. In the top row we used a color map based on the simulation time. The middle and bottom rows used colors to distinguish clusters found respectively by our AI-enhanced model and by the RMSD. Firstly, we noted that the HDBSCAN behaved well in clustering time steps projected into the latent space by the CVAE model, this was noticeable by the agreement of colors mapping in the middle row and actual clusters of points. It was also noticeable that, generally, clusters are formed by time frames close in the simulation. The average *CosSim* between traditional RMSD clustering results and AI-enhanced clustering results was 0.89 ± 0.08 for 5 stable simulations (see Additional file 1: Table S4), and 0.87 ± 0.08 for all 11 simulations. This proved the reliability of our AI-enhance clustering methodology, allowing us to proceed in a fine-grained analysis of the drug molecule behavior. Note that it was not our intention to exactly match traditional RMSD clustering and AI-enhanced clustering. The RMSD clustering method treated the entire molecule as a single point at its center of mass, therefore no rotational or vibrational motions were captured, in contrast to the contact matrices used by AI-enhanced clustering.

In contact matrices, atoms adjacent in columns (from proteins in our case) are often physically close to each other. We tested the reliability of the convolution models by evaluating the clustering results with randomly permuted columns. In Additional file 1: Table S4, we showed that fairly similar clustering results were obtained. This might mean that the proximity of columns was redundant information, because contact matrices should have provided the closeness of atoms already.

### AI-enhanced clustering varies with individual atom subtypes, hinting malicious atom subtypes

We further analyzed the behavior of each atom subtype and compared it to the RMSD reference. With the first bar as reference from RMSD, Fig. 5 offered a visual comparison of the differences in clustering. In particular, each bar was created with a color map that showed the cluster size of each time step *t* of the simulation (with bigger clusters in dark red and smaller in dark blue). The second bar showed the clusters obtained by CVAE and HDBSCAN when using all the sweetener atoms. The remaining bars show the clustering results for each individual subtype. For all the AI-enhanced bars, the averaged cluster size among the clustering results given by the different model trained was showed. All trajectories have a stable binding mode according to RMSD clustering, that showed big red clusters along almost all the simulations. Only sucralose showed a less stable behavior, especially in the final part of the trajectory. As a confirmation of the agreement between RMSD and AI-enhanced clustering, the first two bars of each sweetener agreed well, with an exception of isovanillyl (as was also shown in Fig. 4). The different binding behaviors of the individual subtypes were observable by comparing the subtypes cluster sizes with the RMSD ones. Reddish bars identified stable subtypes, for instance subtype 11 and 27 showed a stable result for sucrose. Inversely, bars with a different coloring with respect to the reference, hinted less stable subtypes that could be marked as “malicious atoms”, because they might weaken the protein-sweetener interactions. In order to quantitatively identify the malicious atoms, we adopted comparison metrics, as reported below.

### Comparison metrics such as *nCosSim* pointed out the malicious atoms

If the sweeteners remained stable in T1R2, the atom subtypes that deviated from the overall stability should be the “malicious atoms”. CASTELO was designed to process MD simulations with **stable** binding modes to find these malicious atoms that weakened T1R2-sweetener binding affinity. We used Eqs. 2 and 3 to compare the stability between the whole molecule (calculated with RMSD clustering) and each of the atom subtypes (calculated with AI-enhanced clustering). The resulting *CosSim* and *AvgDiff* metrics were then normalized by subtracting the average respective metric values (calculated with all atom subtypes). The normalized metric values (*nCosSim* and *nAvgDiff*) were plotted in Fig. 6a from all simulations. In this way, negative *nCosSim* and *nAvgDiff* values indicated that the subgroups were less stable than the overall structure. Positive *nCosSim* and *nAvgDiff* values indicated that the subgroups were more stable than the overall structure. It was not surprising that these two metrics were positively correlated (R$^2$ = 0.31).

It is important to mention that the deviation between atom subtypes and the whole molecule should not be comprehended as a time-averaged measurement such as root mean square fluctuation (RMSF). If we consider an atom subtype that only fluctuates drastically when the whole molecule is unstable, the RMSF of this atom subtype might be high but it does not deviate from the overall behavior of the molecule. Therefore, each of the timeframes must be compared separately between the whole molecule and its atom subtypes. With this in mind, Eqs. 2 and 3 are used as the comparison metrics. Another advantage of AI-enhanced clusters is that they contain rotational and vibrational information, compared to the conventional RMSF calculations that mainly consider translational fluctuations. The additional rotational information from AI-enhanced clusters might be particularly useful to monitor atom groups such as –CH$_3$ and –CH$_2$-that would have been easily ignored otherwise. To clearly show that our metrics differ from conventional RMSF calculations, we compared the *nCosSim* and *nAvgDiff* to RMSF values from all simulations in Fig. 6b, c. Both plots yielded R$^2$ = 0.0, indicating that *nCosSim* and *nAvgDiff* contained independent information from RMSF. To further validate the use of these comparison metrics, we calculated “*dv* scores” [50] that captured the consistency between two clustering results. We plotted the correlation between *dv* scores and *nCosSim* in Additional file 1: Figure S4. Note that higher *dv* scores indicated worse consistency between two clustering methods. Expectedly, there was a negative correlation between *dv* scores and *nCosSim* (R$^2$ = 0.42).

To find the malicious atoms, we focused on the trajectories that comprised largest clusters, such as the ones shown in Fig. 5. This is important because only when the overall simulation is stable, low comparison metric values would mean that the corresponding atoms contributed little to the overall binding. In principle, CASTELO could be used on unstable simulations to pick out beneficial atoms by selecting atoms with high comparison metric values. However, such application is beyond the scope of current study. *nCosSim* and *nAvgDiff* metrics were plotted in Fig. 7 for these trajectories. A ranking system was used to pick out the atom subtype most prone to undermine the overall T1R2-sweetener binding stability. For example, in Fig. 7a, subtype 12 of dulcin was clearly the malicious atom subtype, because it ranked the lowest in both *nCosSim* and *nAvgDiff* metrics. In Fig. 7b, subtype 7 and 8 were mostly likely the malicious atom subtypes, because of their low values in both *nCosSim* and *nAvgDiff*. The selected atom subtypes were chosen as the candidate for lead optimization (see results in the next section).

In Fig. 7c, d, we compared the comparison metrics of sucrose, 4R-Cl-sucrose and sucralose, which could be considered as a lead optimization chain due to their structural similarity. We noticed that the added chlorine atoms (subtype 4) in 4R-Cl-sucrose and sucralose were increasingly beneficial to the overall structural stability (both *nCosSim* and *nAvgDiff* metrics were increasingly positive). The suggested malicious atom subtypes were carbohydrate carbons that connect with hydroxyl groups (–CHOH–, subtype 10). Upon modifying several of the hydroxyl groups to chlorine atoms, both *nCosSim* and *nAvgDiff* metrics of subtype 10 were increasingly more positive, indicating that the replacement of chlorine atoms affected the stability of its connecting carbons. As a comparison, we plotted the relative binding free energy of sucrose, 4R-Cl-sucrose and sucralose in Fig. 7e, which clearly correlated with *nCosSim* and *nAvgDiff* metrics for subtype 4 and subtype 10. Combining this evidence and Table 1, the *nCosSim* and *nAvgDiff* metrics for subtype 4 and subtype 10 correlate well with experimental *RS* values. Finally, we provided a possibility to further improve sucralose by suggesting that the malicious atoms in sucralose were probably from subtype 27, which were the aliphatic hydrogens in the –CH$_2$OH group.

### FEP calculations verified that malicious atom types could be modified to strengthen T1R2-sweetener binding affinity, providing an opportunity for lead optimization

Based on the ranked comparison metric values in Fig. 7a, b, we visualized the malicious atoms of dulcin and isovanillyl in Fig. 8a, b, as a way to guide chemists in lead optimization. For example, subtype 12 of dulcin could be easily changed to a carbonyl (–CO–) or a chloronated carbon (–CHCl–). Subtype 8 of isovanillyl could be easily changed to an aliphatic carbon (–CH$_2$–). In addition to these malicious atoms, we also selected some neutral atoms as control groups (like subtype 40 of dulcin and subtype 10 of isovanillyl). To verify if the identified atoms were indeed “malicious”, FEP calculations were adopted to compare the binding free energy of the modified lead molecules to the lead molecules (exemplified in Fig. 8; a full list could be found in Additional file 1: Table S2 and Table S3). For example, a dual topology was made between dulcin and modified dulcin where group X (also subtype 12, see Additional file 1: Table S2) was changed from CH$_2$ to CHCl. Two sets of simulations were performed: one in T1R2 and one solvated with 0.1 M NaCl solution. The calculated binding free energy (${\Delta }{\Delta }F$) was thus a direct comparison between dulcin and the modified dulcin.

We tried 4 combinations for dulcin optimization varying neutral moiety R and malicious moiety X, shown in Additional file 1: Table S2. Modifications on moiety R always weakened T1R2-dulcin binding affinity. However, modifications on moiety X resulted in one favorable modifications. -2.4 ± 1.0 kcal/mol was obtained when moiety X was changed from –CH$_2$– to –CHCl–. Interestingly, when moiety X was changed to –CO–, the binding affinity was weakened, probably because its surroundings was slightly hydrophobic (I327, seen in Fig. S3E).

Similarly, we attempted 2 combinations for isovanillyl optimization, varying neutral moiety R and malicious moiety X, shown in Additional file 1: Table S3. Modifications on moiety R neither strengthened nor weakened T1R2-isovanillyl binding affinity (${\Delta }{\Delta }F$ was not significant compared to its standard deviation). In contrast, modifications on moiety X significantly strengthened T1R2-isovanilly binding affinity by -3.8 ± 1.0 kcal/mol. It was counter-intuitive that changing moiety X to a hydrophobic group would significantly benefit the binding affinity, given that its surroundings included D142 and Y164 (Additional file 1: Figure S3F). On the other hand, the surroundings of moiety R included V388, F39 and L71, which would likely favor a hydrophobic modification. Further analysis pointed out that by modifying moiety X, its nearby phenol-hydroxyl group was freed from forming intramolecular hydrogen bond and established a hydrogen bond with D142. This transition lowered the electrostatic penalty of charged D142 residue around the hydrophobic isovanillyl (evidenced in Additional file 1: Table S1). Our findings blindly agreed with a previous experimental isovanillyl sweetener optimization which suggested the optimal moiety X to be –CH$_2$–, [28] reinforcing that CASTELO could potentially assist in lead optimization.

Finally, it might be of interest to test the influence of $\delta$ (time difference in calculating the dynamism tensor) on the prediction results. In Additional file 1: Table S5, with dulcin simulation as an example, we showed that *CosSim* between traditional RMSD clustering and AI-enhanced clustering using all atoms remained similar from $\delta$ = 0 to 1000. In Additional file 1: Table S6, we calculated the correlation coefficient between each of the columns in Additional file 1: Table S5 (excluding the first row). We found that the overall correlations seemed to point out that $\delta$ did not affect the predictions of individual atom subtypes much as well. For example, Type12 consistently appeared to have low *nCosSim* values (therefore consistently predicted as a malicious subtype). Differences still occurred, for example in Type23 and Type24. Note that dynamism representation provided additional temporal information, regarding the motions of atoms between *t* and $t-\delta$. If the binding state of a sweetener was stable, the temporal dimension of dynamism matrix of all atoms would give an average of zero motion in space. This might explain why $\delta$ had little impact on the *CosSim* between two clustering methods using **all atoms**. However, dynamism representation might be necessary for atom subtype predictions, because individual atom motions might not be net zero. In the current study, we fixed $\delta$ as 500 as a working example. More studies are needed to fully understand the influence of $\delta$ on the subtype predictions.

## Conclusions

In this work, we introduced CASTELO, a ML-MD pipeline that processed MD simulation data with drug targets and their known leads and suggested modifiable submolecular moieties for lead optimization. We generated dynamism tensors by including temporal information in the conventional contact matrices. CVAE method was adopted to compress the dynamism tensors into latent space before the data clustering with HDBSCAN. The lead molecule was grouped into atom subtypes to pin down submolecular contributions to the target-lead binding affinity. The resulting cluster information was compared to a traditional RMSD-based clustering method for the whole molecule with the proposed comparison metrics *CosSim* and *AvgDiff* . Finally, we ranked the submolecular moieties to find clues for lead optimization. With T1R2-sweetener as a model system, we proved that our pipeline nicely explained the improvement of the sweetness from sucrose to 4R-Cl-sucrose and sucralose. Most notably, we suggested two brand new molecules based on the CASTELO pipeline using T1R2-dulcin and T1R2-isovanillyl simulations. With free energy calculations, we verified that the newly improved dulcin was $\sim$ 57 times sweeter than dulcin, probably $\sim$ 14,000 times sweeter than sucrose. The newly improved isovanillyl was computed to be $\sim$ 600 times sweeter than isovanillyl, probably $\sim$ 240,000 times sweeter than sucrose. Unlike most entirely knowledge-based models, our physics-based model should be transferable to other systems. We plan to use more target-lead systems to test CASTELO’s scalability and interpretability. For example, a similar approach could be adapted for major histocompatibility complex (MHC) and epitope complexes (paper submitted). The identification of any moiety as malicious may indicate destabilizing motions of its surrounding atoms rather than moiety itself. Generative models could be developed on top of the malicious atom identifications in this study. Although CASTELO’s dependency on experimental data is low, we foresee that our tool could be further enhanced with more use cases, especially with more experimental data to reference to. We plan to further investigate the influence of the simulation’s dynamism, by studying the choice of the $\delta$ parameter and also by introducing recurrent neural networks in the AI-enhanced clustering.

## Supplementary Information

**Additional file 1:** **Table S1** provides the binding free energy decomposition on the T1R2-sweetener systems. **Table S2** is provided for the *in silico* lead optimization of dulcin. **Table S3** is provided for the *in silico* lead optimization of isovanillyl. **Table S4** lists the comparison metric results when all columns of contact matrices are randomly permuted. **Table S5** lists the prediction results of dulcin with $\delta$ as a variable (between 0 to 1000). Table S6 shows the correlation between the predictions at different $\delta$ values. **Figure S1** is attached to show the thermodynamic cycle used for FEP calculations. **Figure S2** plots the stable clusters identified by RMSD clustering. **Figure S3** illustrates the stable binding structures for the five sweeteners in the T1R2 flytrap domain. **Figure S4** depicts the correlation between *CosSim* and *dv* scores.

## Data Availability

The datasets generated and/or analysed during the current study are not publicly available due to the agreement between IBM and a confidential client but are available from the corresponding author on reasonable request, with the permission of the client (which will remain confidential).
