# Supplementary material for: CASTELO: clustered atom subtypes aided lead optimization—a combined machine learning and molecular modeling method
Source: BMC Bioinformatics. 2021 Jun 22;22:338. doi: 10.1186/s12859-021-04214-4 (PMC8218488; doi:10.1186/s12859-021-04214-4)
Supplement: Supplementary file 1 — Additional file 1: Table S1 provides the binding free energy decomposition on the T1R2-sweetener systems. Table S2 is provided for the in silico lead optimization of dulcin. Table S3 is provided for the in silico lead optimization of isovanillyl. Table S4 lists the comparison metric results when all columns of contact matrices are randomly permuted. Table S5 lists the prediction results of dulcin with $\delta$δ as a variable (between 0 to 1000). Table S6 shows the correlation between the predictions at different $\delta$δ values. Figure S1 is attached to show the thermodynamic cycle used for FEP calculations. Figure S2 plots the stable clusters identified by RMSD clustering. Figure S3 illustrates the stable binding structures for the five sweeteners in the T1R2 flytrap domain. Figure S4 depicts the correlation between CosSim and dv scores. [file 12859_2021_4214_MOESM1_ESM.pdf]

SUPPLEMENTARY MATERIALS
CASTELO: Clustered Atom Subtypes aidEd Lead Optimization - a combined machine learning and molecular modeling method

Leili Zhang, Giacomo Domeniconi, Chih-Chieh Yang, Seung-gu Kang, Ruhong Zhou, Guojing Cong

Table S1 provides the binding free energy decomposition on the T1R2-sweetener systems. Table S2 is provided for the *in silico* lead optimization of dulcin. Table S3 is provided for the *in silico* lead optimization of isovanillyl. Figure S1 is attached to show the thermodynamic cycle used for FEP calculations. Figure S2 plots the stable clusters identified by RMSD clustering. Figure S4 illustrates the stable binding structures for the five sweeteners in the T1R2 flytrap domain.

Sweetener	$\Delta\Delta F$	$\Delta\Delta F_{elec}$	$\Delta\Delta F_{vdw}$
Sucrose	-6.9 ± 0.7	-8.0 ± 0.5	0.2 ± 0.5
4R-Cl-sucrose	-10.2 ± 0.8	-9.3 ± 0.5	-2.3 ± 0.6
Sucralose	-11.7 ± 0.8	-11.5 ± 0.6	-1.9 ± 0.6
Dulcin	-10.6 ± 0.4	-7.1 ± 0.3	-3.2 ± 0.6
Isovanillyl	-11.1 ± 0.8	0.8 ± 0.5	-12.8 ± 0.6

Tab. S1: Binding free energy decomposition. $\Delta\Delta F_{elec}$ or $\Delta\Delta F_{vdw}$ is calculated with Zwanzig equation when only electrostatic energy or VDW energy is considered. Note that due to the construction of Zwanzig equation, $\Delta\Delta F$ is not a simple addition of $\Delta\Delta F_{elec}$ or $\Delta\Delta F_{vdw}$.

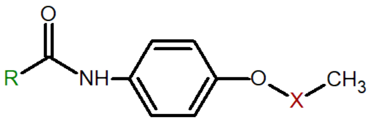

R	X	$\Delta\Delta F$
NH_2	CH_2	0
NH_2	CO	+2.5 ± 1.1
NH_2	CHCl	-2.4 ± 1.0
CH_3	CH_2	+2.4 ± 1.3
$NHCH_2CH_3$	CH_2	+4.9 ± 1.3

Tab. S2: Lead optimization for dulcin. Relative free energy $\Delta\Delta F$ is calculated using the original dulcin molecule as the reference (where R is NH_2 and X is CH_2). Negative values of $\Delta\Delta F$ indicate that the modifications strengthen the binding affinity. The unit of $\Delta\Delta F$ is kcal/mol.

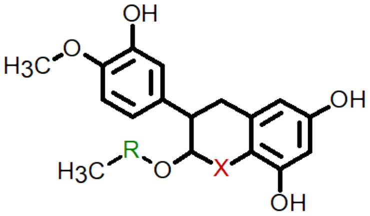

R	X	$\Delta\Delta F$
CO	CO	0
CO	CH_2	-3.8 ± 1.0
CH_2	CO	-1.0 ± 1.4

Tab. S3: Lead optimization for isovanillyl. Relative free energy $\Delta\Delta F$ is calculated using the original isovanillyl molecule as the reference (where R and X are both CO). Negative values of $\Delta\Delta F$ indicate that the modifications strengthen the binding affinity. The unit of $\Delta\Delta F$ is kcal/mol.

AI-enhanced clustering vs RMSD					
	Sucrose	4R-Cl-sucrose	Sucralose	Dulcin	Isovanillyl
$CosSim$	0.975	0.871	0.785	0.96	0.882
$AvgDiff$	-0.263	-0.814	-0.209	-0.201	-0.65
Δ of results from permuted matrices					
	Sucrose	4R-Cl-sucrose	Sucralose	Dulcin	Isovanillyl
$\Delta CosSim$	0.007	-0.002	-0.006	0.013	-0.01
$\Delta AvgDiff$	0.041	-0.072	0.012	0.052	0.05

Tab. S4: The top table shows the $CosSim$ and $AvgDiff$ of our AI-enhanced clustering compared to the conventional RMSD clustering. The bottom table shows Δs computed as difference between the cosine similarity, and average difference, obtained from the CVAE models when fed by the original contact matrix (as in the top table) and by a version with randomly permuted columns. The Δs' uncertainty proves that the columns order does not affect the reliability of the final clustering.

Residue	$\delta = 0$	$\delta = 100$	$\delta = 250$	$\delta = 500$	$\delta = 1000$	Subtype description
original*	0.933	0.950	0.949	0.960	0.947	All atoms
Type07	0.021	0.017	0.013	-0.004	-0.055	Benzene/urea carbons
Type10	0.009	0.009	-0.010	0.019	0.031	Ether oxygens
Type12	-0.026	-0.048	-0.019	-0.074	-0.002	C in CH2
Type13	0.005	0.025	0.018	-0.005	0.034	C in CH3
Type23	-0.041	-0.019	-0.021	0.012	-0.078	Carbonyl oxygen
Type24	-0.054	-0.015	-0.041	0.006	-0.017	Urea hydrogens
Type25	0.032	0.025	0.016	0.015	0.023	H in CH3
Type27	0.033	-0.009	0.028	0.026	0.020	H in CH2
Type28	0.028	0.023	0.020	0.008	0.021	Benzene hydrogens
Type40	-0.008	-0.008	-0.005	-0.003	0.023	Urea nitrogens

Tab. S5: Consistency test with dynamism time interval δ as variable. All data shown (except "original") are normalized cosine similarities ($nCosSim$) between RMSD clustering method and AI enchanced clusering method calculated from the simulation with dulcin with the exception of "original". Values lower than -0.04 were colored with dark red; values lower than -0.015 were colored with light red. *$CosSim$ calculated with all atoms ("original") was not normalized.

δ	0	100	250	500	1000
0	1	0.47	0.87	0.12	0.24
100		1	0.42	0.35	0.11
250			1	0.09	0.17
500				1	0.01
1000					1

Tab. S6: We calculated the linear correlation coefficient R^2 values between each of the columns in Table S5. Note that the matrix is symmetrical. So for simplicity, half of the matrix is hidden.

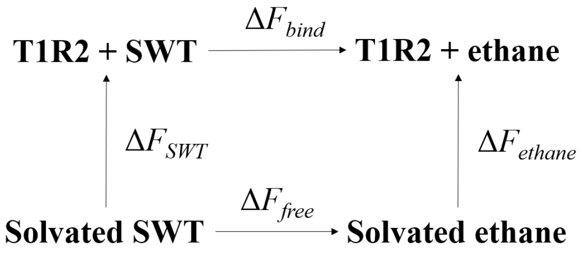

Fig. S1: The thermodynamic cycle for FEP calculations. See method for details.

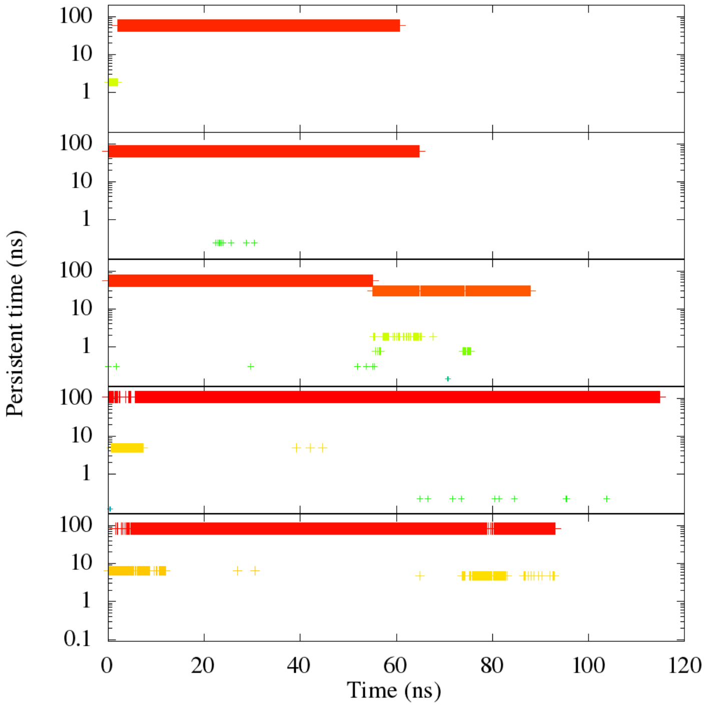

Fig. S2: From the MD simulations, we identify the clusters of sweetener binding states with RMSD calculations on the sweetener alone, while the structure of T1R2 is aligned. The cutoff for the RMSD clustering method is 2 Å. The clusters are plotted using their "persistent time" (defined as the total time each cluster persists in the corresponding simulation). From top to bottom, we plot the persistent time of sucrose, 4R-Cl-sucrose, sucralose, dulcin and isovanillyl, respectively.

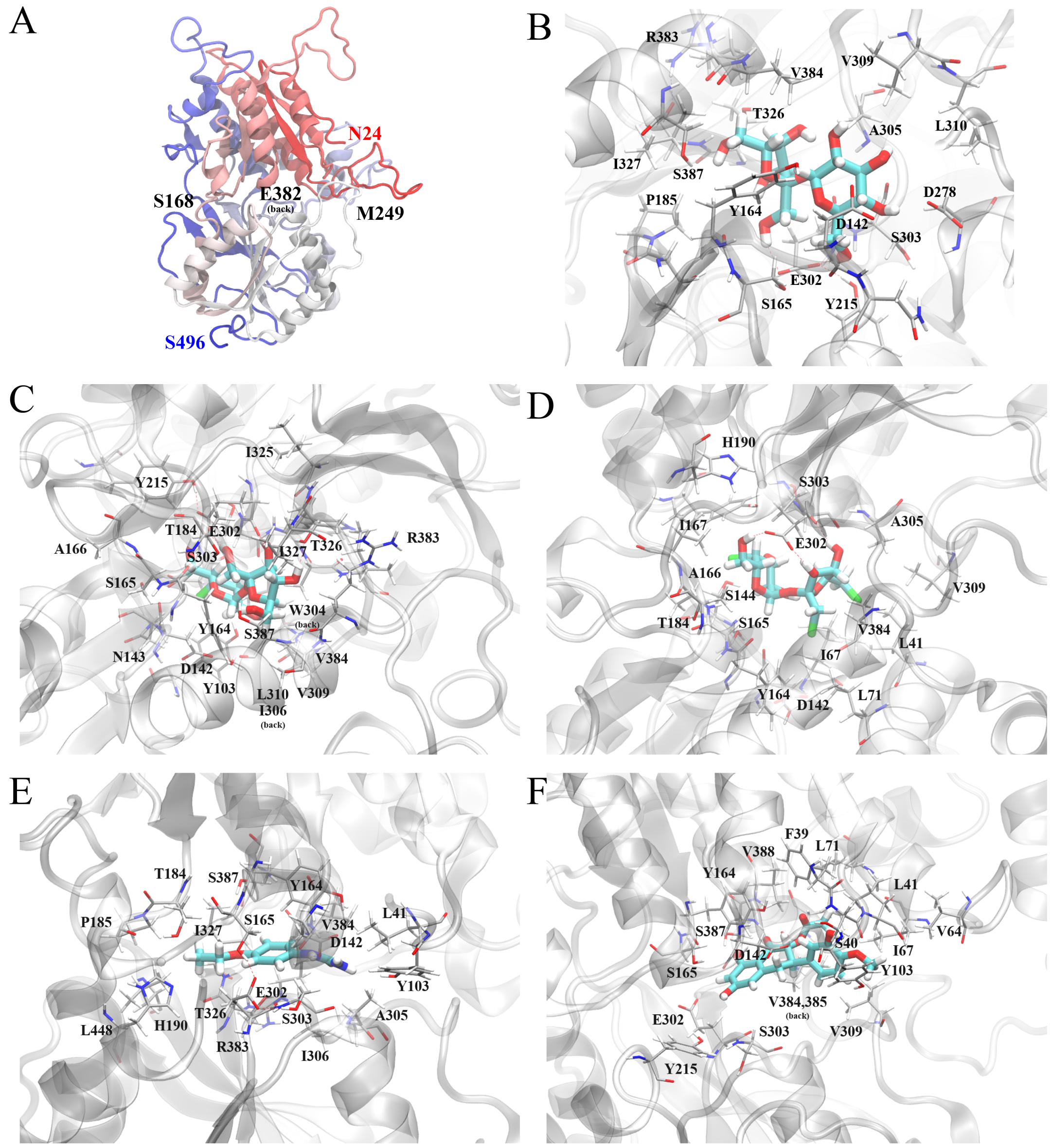

Fig. S3: (A) We illustrate T1R2 protein structure with N-terminus colored by red and C-terminus colored by blue. Stable binding structures of the five sweeteners in T1R2 are identified from the MD simulations and drawn in (B) with sucrose, (C) with 4R-Cl-sucrose, (D) with sucralose, (E) with dulcin and (F) with isovanillyl, respectively. Protein structures are drawn with transparent “newcartoon” representation in VMD. Sweeteners and their interacting residues are drawn with sticks models. The colors for the sticks models are selected as follows: cyan for carbon in the sweeteners, grey for carbon in T1R2, white for hydrogen, red for oxygen, blue for nitrogen, and green for chlorine.

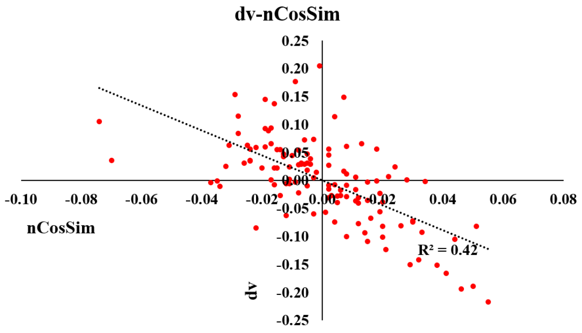

Fig. S4: The correlation between dv scores (Gionis et al.) and *nCosSim*. All metrics were calculated by comparing the clusters with conventional RMSD clustering and AI-enhanced clustering with all 11 T1R2-sweetener simulations. R^2 is calculated to be 0.42.
